# The burden of common mental disorders and their association with diabetes in rural Bangladesh: findings from a population-based cross-sectional study

**DOI:** 10.7189/jogh.15.04220

**Published:** 2025-08-08

**Authors:** Malini Pires, Carina King, Sanjit Shaha, Abdul Kuddus, Naveed Ahmed, Joanna Morrison, Andrew Copas, Sarker Ashraf Uddin Ahmed, Tasmin Nahar, Hassan Haghparast-Bidgoli, Kishwar Azad, Edward Fottrell

**Affiliations:** 1 UCL Institute for Global Health, University College London, London, UK; 2 Department of Global Public Health, Karolinska Institutet, Stockholm, Sweden; 3 Centre for Health Research and Implementation, Diabetic Association of Bangladesh, Dhaka, Bangladesh

## Abstract

**Background:**

Type 2 diabetes mellitus (T2DM) and common mental disorders (CMDs), including depression and anxiety, are significant public health challenges, particularly in low- and middle-income countries (LMICs). However, evidence on the distribution and associations between T2DM and CMDs in rural LMIC populations remains limited. We aimed to examine this relationship in a sample of adults from rural Bangladesh.

**Methods:**

In this cross-sectional study, we analysed baseline data from a cluster-randomised controlled trial conducted in rural Faridpur, Bangladesh in 2021. A total of 1392 randomly sampled adults aged ≥30 years participated in the original trial. Here, we identified T2DM through fasting glucose levels and post-glucose load results, or self-reported healthcare diagnosis. We assessed depression and anxiety using the Bangla versions of the Patient Health Questionnaire and Generalized Anxiety Disorder 7 scales. Among others, we also collected data on sociodemographic factors. We assessed the prevalence of different CMDs and T2DM, and estimated the association between the two through multivariate logistic regression adjusted for sociodemographic variables.

**Results:**

We found a prevalence of 6.0% for depression, 4.0% for anxiety, and 2.2% for the comorbid depression and anxiety. Compared to those without T2DM, participants with T2DM had higher odds of having depression (adjusted odds ratio (aOR) = 1.93; 95% confidence interval (CI) = 1.37–2.73) and comorbid depression and anxiety (aOR = 1.99; 95% CI = 1.13–3.50). No significant association was found between T2DM and anxiety (aOR = 1.38; 95%CI = 0.87–2.19). Age, marital status, and employment also influenced CMD risk.

**Conclusions:**

There is a significant association between T2DM, depression, and comorbid depression and anxiety in rural Bangladesh, with gender potentially playing a modifying role. Integrated, gender-sensitive care models addressing both mental health and diabetes are essential in rural healthcare settings.

Multimorbidity, the presence of two or more chronic conditions in one individual [1], is increasingly recognised as a significant public health challenge that worsens health outcomes, strains healthcare systems, and imposes a heavy burden on patients through increased healthcare costs, reduced quality of life, and elevated morbidity and mortality rates [2]. Key factors driving multimorbidity include ageing populations, shifts in lifestyle habits, and poverty [3,4].

The global multimorbidity-related burden is primarily influenced by type 2 diabetes mellitus (T2DM) [4,5]. Estimates suggest that 537 million adults globally were living with diabetes in 2021, with projections suggesting this number could increase to 643 million by 2030 [5]. Approximately 90% of these cases have been attributed to T2DM [5]. The global burden of disability-adjusted life-years attributable to T2DM more than doubled from 1990 to 2019 [6]. Approximately 80% of diabetes cases occur in low- and middle-income countries [5]. In South Asia, the prevalence of T2DM has surged alongside other non-communicable diseases due to rapid demographic, epidemiological, and socioeconomic changes [7]. As of 2021, Bangladesh reported a diabetes prevalence of 14.2% – an increase from 10.7% in 2011 – with an estimated 13.1 million adults affected [5]. A review from 2016 which pooled data from 22 studies estimated diabetes prevalence to be 7.4% (95% confidence interval (CI) = 7.2–7.7), with men having a higher prevalence in urban areas and women in rural areas [8].

The Global Burden of Disease Study in 2019 reported a prevalence of 3.8% for depressive disorders and 3.0% for anxiety disorders in South Asia [6]. In Bangladesh, a nationwide survey conducted between 2003 and 2005 estimated the prevalence of all mental disorders to be 16.1% [9], while subsequent research in 2019 found it to be 18.7% [10]. The latter study reported the prevalence of depressive disorders to be 6.7% and anxiety disorders to be 4.5% [10]. This gives cause for concern, as mental healthcare in Bangladesh is inadequate, with the government spending on mental health being approximately 0.4% of the total health budget, and as less than 0.11% of the population has access to free medication for mental health disorders [11].

Common mental health disorders have not been always considered as contributors to multimorbidity [2]. However, studies have shown that people with T2DM are twice as likely to have depression compared to those without, with these two disorders causing an incrementally negative impact on health outcomes [12,13]. Common mental disorders significantly impair quality of life and are associated with increased morbidity. In 2019, depression was the second leading global cause of years lived, with disability and anxiety ranked eighth [6].

For people with T2DM in India, Pakistan, and Bangladesh, the prevalence of depression was estimated to be 40% (95% CI = 34–45), while that of anxiety was estimated at 29% (95% CI = 16–44) [14]. Given the high prevalence of both T2DM and mental health disorders in this region, there is a pressing need for studies of their association. In the context of Bangladesh specifically, most research on this topic has been conducted in clinical settings in urban areas, although approximately 61% of the country’s population resides in rural areas [15]. To address these gaps, we aimed to describe and explore associations between T2DM status and mental health conditions (depression, anxiety, and comorbid depression and anxiety) in rural Bangladesh.

## METHODS

We utilised baseline data collected in a cross-sectional survey from January to March 2021 from the Diabetes Community-Led Awareness, Response and Evaluation (D:Clare) cluster-randomised controlled trial conducted in rural Faridpur, Bangladesh, focussing on T2DM prevention and control (registration: ISRCTN42219712) [16,17]. While the D:Clare trial’s primary objective was to evaluate the effectiveness of an intervention on T2DM-related outcomes, with subsequent publications addressing aspects of diabetes prevalence, risk factors, and intervention strategies in rural Bangladesh [16,17], we focussed exclusively on examining the association between T2DM status and the prevalence of common mental disorders (CMDs), *i.e.* depression, anxiety, and comorbid depression and anxiety, within this population.

We adhered to the STROBE guidelines for observational studies [18].

### Setting

We conducted this study in Alfadanga *upazila* in the Faridpur district [17]. Bangladesh is administratively divided into divisions, districts (*i.e.* *zilas*), and sub-districts (*i.e.* *upazilas*). Rural areas within *upazilas* are further divided into unions, which represent the lowest rural administrative unit. Alfadanga, comprised of six unions, is characterised by a predominantly agricultural economy and is officially designated as a rural area [19]. Faridpur is divided into nine *upazilas*, which in turn are divided into unions of approximately 25 000 population per union [20]. Faridpur district is situated on the banks of the Padma River and spans an area of 2050 km^2^, with a population of over 1.7 million and a predominantly agricultural economy. The population is primarily Bengali, with around 90% identifying as Muslim and approximately 10% as Hindu [16].

Healthcare services in Bangladesh are provided by the government, private sector, and non-governmental organisations. Public healthcare is delivered through district hospitals, sub-district health complexes, union-level health and family welfare centres, and community clinics at the ward level. Specialised diabetes care is offered by the Bangladesh Diabetic Association, with facilities in every district [21].

### Sampling

We used multi-stage sampling, dividing six unions in Alfadanga into 12 clusters. To minimise the risk of contamination between intervention and control clusters (for the D:Clare trial), we purposefully selected central villages, excluding those on cluster borders, major trading centres, or administrative hubs. We selected between two and five villages with at least 50 households in each cluster to achieve a goal of 800–1000 households per cluster. We used the 2011 Bangladesh census and administrative maps to estimate cluster population sizes. A household census in November 2019 generated a sampling frame from which we randomly selected 132 households in each cluster. In each selected household, we randomly chose one eligible adult (aged ≥30 years, residing in Alfadanga for at least six months) to participate [16,17].

### Data collection

Locally hired trained data collectors conducted data collection, with anthropometric data collected and a household survey adapted from the World Health Organizations’ Stepwise tool and questions from the 2014 Bangladesh Demographic and Health Survey [19,22]. They obtained whole blood by finger prick and measured blood glucose levels in mmol/L and tested the participants after an overnight fast of 8–12 hours and then again two hours after receiving a 75 g glucose load dissolved in 250 ml of water.

The collected data included sociodemographic and socioeconomic variables (age, sex, marital status, religion, education level, respondent’s occupation, and occupation of head of household). The data collectors gathered the information on household assets and income to calculate socioeconomic status. They used the nine-item Patient Health Questionnaire (PHQ-9) to assess the existence and severity of depressive symptoms [23]. Specifically, we evaluated patients who screened positive in the PHQ-2 (the first two items of the PHQ-9 tool) [24] with the full PHQ-9 to determine the severity of depressive symptoms. To screen for anxiety, we used the Generalized Anxiety Disorder 7-item (GAD-7) screening tool [23,25], the Bangla version of which had been previously validated and used it in the Bangladeshi context [26,27].

The interviewer collected the data using ODK Collect, version 1.26 (ODK Inc., San Diego, USA), which was then uploaded to a central server by the D:Clare team on a weekly basis for routine cleaning and checks.

### Analysis

The main independent variable of interest in this study was T2DM status. The outcome variables were the prevalence of depression, anxiety, and comorbid depression and anxiety. We defined a person with T2DM as having a fasting plasma glucose of ≥7.0 mmol/L, a two-hour post-glucose load blood glucose of ≥11.1 mmol/L, or a self-reported diagnosis of diabetes by a healthcare provider [5].

For depression, we categorised PHQ-9 scores into a binary variable (no depression or depression), whereby we categorised all participants who were screened for the PHQ-2 and scored <3 as not having depression. Of the participants who scored ≥3 on the PHQ-2 and went on to be screened with the PHQ-9, we categorised those who scored <10 (no depressive symptoms or mild depression) as being without depression and those with PHQ-9 scores of ≥10 (moderate or high depression) as having depression. This cut-off is commonly used in the literature and balances between the questionnaire’s sensitivity and specificity [28].

We also categorised anxiety into a binary variable, defining patients with GAD-7 scores <10 as having no anxiety and those with scores ≥10 as having anxiety. The literature shows that a GAD-7 score of ≥10 is a suitable threshold for identifying individuals with generalised anxiety disorder [25]. We created a binary variable for comorbid depression and anxiety if a participant scored ≥10 in both the PHQ-9 and GAD-7.

### Descriptive analysis

We conducted a descriptive analysis of the sociodemographic and socioeconomic characteristics of the survey participants and estimated the prevalence of T2DM, depression, anxiety, and comorbid depression and anxiety.

To assess household wealth, we constructed a standard of living index following methods used in Demographic and Health Surveys [29]. This involved using principal component analysis to generate a composite wealth score based on asset ownership (*e.g.* mobile phone, television, bicycle), access to electricity, quality of housing materials (*e.g.* walls), land ownership, and sanitation facilities. We coded variables as binary indicators of deprivation. We used the first principal component to create a continuous wealth score, which we then divided into tertiles to represent relative household wealth (*i.e.* most poor, poor, and least poor). We kept age as a continuous variable. We also analysed other sociodemographic variables, including were sex, marital status, occupation (where paid employment was defined as having an occupation), and level of education (Table S1 in the **Online Supplementary Document**).

### Multivariate logistic regression

We used multivariate logistic regression to assess the association between T2DM and outcomes, adjusted for the sociodemographic variables described to account for possible confounding [30,31]. After the initial analysis, we explored the potential modifying effect of sex on the relationship between T2DM and CMD outcomes through stratified multivariate logistic regression. This was based on evidence of gender differences in health and social factors that could influence the association of T2DM and CMDs in Bangladesh [32–34]. We also tested interaction terms between T2DM and sex to determine whether sex significantly influenced the association for each CMD outcome (depression, anxiety, and comorbid depression and anxiety).

All analyses accounted for the clustered survey design and weighting for the unequal probability of selection, and were performed using the complex survey ‘svy’ functions in STATA, version 18 (StataCorp LLC, College Station, Texas, USA).

### Ethics

Written informed consent was obtained from all participants before data collection, or a thumbprint for those who were illiterate or unable to write. All data were anonymised before analysis, and access was restricted to the research team to ensure participant confidentiality.

## RESULTS

Of the target sample of 1584 individuals, 1392 (87.9%) participated in the survey. Non-participation was primarily attributed to temporary and permanent migration, death, or lack of interest. We described the sample by diabetes status, sociodemographic characteristics, and mental health status, specifically depression, anxiety, and comorbid depression and anxiety (**Table 1**). In short, a total of 242 (17.4%; 95% CI = 14.3–21.0) participants had diabetes, 84 (6.0%; 95% CI = 2.5–13.5) had depression, 60 (4.0%; 95% CI = 1.9–8.0) anxiety, and 33 (2.2%; 95% CI = 0.8–5.8) had comorbid depression and anxiety.

**Table 1:** Summary of diabetic status, sociodemographic characteristics, and mental health status of participants*

	n (%)
**Total number of participants**	1392
**Diabetes†**	
No diabetes	1137 (82.6)
Diabetes	242 (17.4)
**Sex**	
Male	516 (40.0)
Female	876 (60.3)
**Age, x̄ (SE)**	50.9 (0.67)
**Marital status**	
Married	1182 (86.8)
Not married	210 (13.2)
**Income tertile**	
Poorest	465 (32.5)
Poor	463 (33.8)
Least poor	464 (33.7)
**Occupation**	
Has occupation	467 (34.0)
No occupation	925 (66.0)
**Education**	
Education	568 (39.1)
No education	824 (61.0)
**Depression**	
No depression	1308 (94.0)
Depression	84 (6.0)
**Anxiety**	
No anxiety	1332 (96.0)
Anxiety	60 (4.0)
**Comorbid depression and anxiety**	
No depression and anxiety	1359 (97.8)
Depression and anxiety	33 (2.2)

SE – standard error, x̄ – mean

*Values presented as absolute numbers (n) and weighted percentages (%) unless specified otherwise.

†Thirteen participants missing data.

### Depression

When adjusting for sex, age, marital status, income, and occupation, the adjusted odds ratio (aOR) indicated that the odds of having depression among people with diabetes were 1.93 times higher (95% CI = 1.37–2.73, *P* < 0.001) compared to people without diabetes (**Table 2**).

**Table 2:** Weighted ORs and aORs for the association of T2DM and sociodemographic variables with depression (n = 1392)

	Total n	Depression, n (%)	OR (95% CI)	*P*-value	aOR (95% CI)	*P*-value
Diabetic status*						
No diabetes	1137	62 (5.4)	ref		ref	
Diabetes	242	22 (8.9)	1.71 (1.26–2.33)	0.003	1.93 (1.37–2.73)	<0.001
**Sex**						
Male	516	23 (4.8)	ref		ref	
Female	876	61 (6.8)	1.45 (0.90–2.37)	0.117	0.89 (0.33–2.39)	0.799
**Age**	1392		1.06 (1.03–1.08)	<0.001	1.05 (1.02–1.07)	<0.001
**Marital status**						
Married	1182	33 (4.4)	ref		ref	
Not married	210	51 (16.0)	4.12 (2.15–7.89)	0.001	1.89 (0.97–3.67)	0.058
**Income tertile**						
Poorest	465	34 (6.2)	ref		ref	
Poor	463	28 (6.9)	1.12 (0.75–1.66)	0.543	1.17 (0.83–1.65)	0.350
Least poor	464	22 (4.8)	0.77 (0.34–1.66)	0.476	0.64 (0.20–2.00)	0.408
**Occupation**						
Has occupation	467	12 (2.3)	ref		ref	
No occupation	925	72 (7.8)	3.51 (1.88–6.55)	0.001	2.96 (1.15–7.60)	0.028
**Education**						
Education	568	24 (3.9)	ref		ref	
No education	824	60 (7.3)	1.95 (1.01–3.76)	0.047	0.89 (0.31–2.60)	0.820

aOR – adjusted odds ratio, CI – confidence interval, OR – crude odds ratio, ref – reference, T2DM – type 2 diabetes mellitus

*Thirteen participants missing data.

For age, the results showed that for each year increase in age, there is a 5% increase in the odds of having depression (aOR = 1.05; 95% CI = 1.02–1.07, *P* < 0.001). Respondents who were not married had 1.89 times higher odds (95% CI = 0.97–3.67, *P* = 0.058) of having depression compared to those who were married. Those without a formal occupation had almost a three-fold increase in the odds of having depression compared to those with a formal occupation (aOR = 2.96; 95% CI = 1.15–7.60, *P* = 0.028). When adjusted for other demographic characteristics, sex, income, and education showed no association with depression.

*Post-hoc* stratification showed that there were differences in the association between women and men. The aOR was 2.18 (95% CI = 1.22–3.91, *P* = 0.013) in women compared to 1.25 (95% CI = 0.42–3.67, *P* = 0.656) in men (**Table 3**). However, there was no evidence of interaction.

**Table 3:** Association between depression and diabetes when stratified by sex*

	Depression, n (%)	No depression, n (%)	aOR (95% CI)	*P*-value
**Non-stratified (n = 1378)**				
Diabetes	22 (8.9)	220 (91.1)		
No diabetes	62 (5.4)	1075 (94.6)	1.93 (1.37–2.73)	0.001
**Male (n = 514)**				
Diabetes	5 (6.3)	79 (93.7)		
No diabetes	18 (4.6)	412 (95.5)	1.25 (0.43–3.67)	0.656
**Female (n = 865)**				
Diabetes	17 (10.6)	141 (89.4)		
No diabetes	44 (6.1)	663 (94.0)	2.18 (1.22–3.91)	0.013

aOR – adjusted odds ratio, CI – confidence interval

*Adjustments were made for age, income, education, occupation, and marital status. The analysis did not show evidence of interaction.

### Anxiety

Univariate and multivariate logistic regression (adjusted for sex, age, marital status, income, occupation, and education) showed no significant association between diabetes and anxiety (Table S2 in the **Online Supplementary Document**). However, women had three times higher odds of having anxiety compared to men (aOR = 3.34; 95%CI = 1.30–8.61, *P* = 0.017). The multivariate logistic regression also showed that for every additional year of age, individuals had 4% higher odds of having anxiety (aOR = 1.04; 95% CI = 1.02–1.07, *P* = 0.006). No significant association was found between marital status, income tertile, occupation, and education and the existence of anxiety.

The original (unstratified) aOR for the association between diabetes and anxiety was 1.38 (95% CI = 0.87–2.19, *P* = 0.156). Stratifying by sex did not show a statistically significant association between diabetes and anxiety (Table S3 in the **Online Supplementary Document**).

### Comorbid depression and anxiety

People with diabetes had 1.99 times higher odds of having comorbid depression and anxiety compared to those without diabetes (aOR = 1.99; 95% CI = 1.13–3.50, *P* = 0.022). A yearly increase in age increased the odds of having comorbid depression and anxiety by 5% (aOR = 1.05; 95% CI = 1.01–1.10, *P* = 0.015) (Table S4 in the **Online Supplementary Document**). Univariate logistic regression showed a significant association between sex, marital status, and occupation, with comorbid depression and anxiety. However, after adjusting for other sociodemographic variables, no significant difference was observed.

After stratification by sex, the associations are similar between men (aOR = 1.59; 95% CI = 0.25–10.13, *P* = 0.592) and women (aOR = 1.78; 95% CI = 0.99–3.19, *P* = 0.054) (Table S5 in the **Online Supplementary Document**).

## DISCUSSION

We aimed to investigate the prevalence of depression, anxiety, and comorbid depression and anxiety among individuals with and without T2DM in rural Bangladesh, and to examine their associations with key sociodemographic factors. We found that people with diabetes had significantly higher odds of experiencing depression and comorbid depression and anxiety, but no significant association was observed with anxiety alone. Additionally, factors such as older age, being unmarried, and unemployment were consistently associated with an increased likelihood of depression and comorbid mental health conditions. Notably, a *post-hoc* analysis stratified by sex showed a significant association between diabetes and depression in women, but not in men.

The burden of depression among people with diabetes in our study was 8.9%, compared to 5.4% among those without diabetes. However, a 2004 study in an urbanising area north of Dhaka reported higher rates of depression (29.7% in individuals with diabetes and 14.1% without diabetes) based on the Montgomery and Aasberg Depression Rating Scale with a cut-off score of ≥20 [35]. A follow-up 2009 study in the same area that used a lower cut-off score of ≥13 found similarly elevated rates (31.6% in individuals with diabetes and 15.3% without diabetes) [36]. Differences in diagnostic tools, cut-off scores, and contextual factors likely contribute to the variations observed across studies.

One could argue that using screening tools like the World Health Organization’s Self-Reporting Questionnaire, which groups depressive, anxiety, and psychosomatic symptoms as evidence of common mental disorders or mental distress, would be more appropriate for data collectors with limited formal training [37]. However, for this study, it was essential to assess depression and anxiety separately, as we focussed on their distinct associations with T2DM, given their differing impacts. Evidence from South Asia supports the validity and reliability of the PHQ-9 and GAD-7 for rural populations, including those with or at risk of diabetes, whereby a study from the Kerala Diabetes Prevention Program found these tools performed comparably to those in high-income countries, demonstrating stability across groups such as age, sex, and education [38].

The bidirectional relationship between diabetes and depression is well established in the literature, with biological mechanisms (*e.g.* inflammation, hormonal imbalances) and behavioural factors (*e.g.* poor medication adherence, lifestyle changes) playing central roles [39–43]. A meta-analysis demonstrated an increased risk of depression among people with diabetes, with subgroup analyses showing variability based on study design and geographic location, highlighting the complexity of the diabetes-depression relationship and the importance of context-specific interpretations [44].

### Sociodemographic associations with T2DM and CMDs

Stratified analyses suggested a stronger association between T2DM and depression in women compared to men, though this was not a primary focus of the study. This highlights a need for further research into potential gender differences in the burden of diabetes and mental health.

Additionally, older age, being unmarried, and unemployment were associated with higher odds of depression, irrespective of diabetic status. This emphasises the influence of social and demographic factors, as well as a need for holistic approaches tailored to gender that address both the psychological and social dimensions of care across populations.

Research reports that lower levels of social support, social participation, and financial support in older adults are linked to decreased overall well-being and increased depressive symptoms [45,46]. Social participation may have a protective effect, having been associated with lower odds for depression in older adults, particularly in older women [47]. Additionally, family dynamics play a critical role in managing T2DM, where supportive interactions can significantly enhance self-management and coping [48].

It is also well documented that chronic conditions and multimorbidity, which become increasingly prevalent with advancing age, can have detrimental impacts on health-related quality of life and mental health [49]. One can therefore assume that if there is decreased support and social engagement as the person ages (*e.g.* with widowed women), it will contribute to worsening well-being [50].

In our analysis, both univariate and multivariate logistic regression models indicated no significant link between anxiety and T2DM. There was a strong association between anxiety and older people compared to younger people. Factors related to this are like those reported with depression, where diminishing social networks, loneliness, and increased morbidity could be major contributing factors [12,51].

### Implications for healthcare delivery

Our findings have practical implications for rural healthcare systems in low- and middle-income countries such as Bangladesh. The significant association between T2DM and depression, as well as comorbid depression and anxiety, highlights the importance of integrated care approaches that bridge physical and mental health services [11]. In resource-constrained settings, where access to specialist care is limited, task-shifting strategies that equip community health workers and primary care providers with basic skills to recognise or screen for depression and anxiety symptoms and provide brief psychosocial intervention. This, alongside routine diabetes care, could enhance both physical and mental health detection and outcomes [21,52].

Embedding mental health services into existing non-communicable disease-care healthcare pathways could improve self-management, adherence to treatment, and quality of life for individuals with T2DM. Strengthened referral systems and community-based psychosocial support may also help create more holistic and patient-centred care models in underserved rural areas [53].

This study had three key limitations. First, given its cross-sectional nature, we cannot establish a causal relationship between diabetes and mental health outcomes. Future longitudinal studies are needed to confirm the directionality of this association. Second, although this was a relatively large survey, the number of individuals meeting criteria for depression, anxiety, and comorbid conditions was small. This likely reduced our statistical power to detect associations, particularly for anxiety, and may have resulted in type II errors. Third, although we used validated translated tools (PHQ-9, GAD-7), the cultural expression of psychological distress in rural Bangladesh often takes somatic forms and is expressed in local terms rather than their literal translations [54]. This may reduce the sensitivity of tools developed in high-income contexts, potentially leading to under-identification of anxiety. Unmeasured confounding variables, such as perceived stigma, caregiving burden, or religious coping, may also have influenced our findings [54]. Future longitudinal or mixed-methods studies that account for these factors may help clarify the nature of the T2DM-anxiety relationship in this setting. Additionally, while we have analysed multimorbidity, our focus has been primarily on the associations between diabetes, depression, and anxiety. This approach provides valuable insights into a critical pattern of multimorbidity. However, the scope of morbidities examined is limited, and we acknowledge that more complex patterns of multimorbidity are likely to exist, involving other conditions such as respiratory and cardiovascular diseases, which were not explored in this analysis [3,4]. Future work could explore these broader patterns to gain a more comprehensive understanding of the relationships between these conditions in this setting.

## CONCLUSIONS

This study fills a gap in the literature by describing the current burden of depression, anxiety, and comorbid depression and anxiety, as well as their association with T2DM in rural Bangladesh. Because almost two-thirds of the population still reside in deprived and underserved rural populations, it is crucial to highlight the magnitude of the problem. Further research is needed in rural Bangladesh to generate data that informs resource allocation for integrated health services. Strengthening community-based screening, prevention, and mental health support systems can enhance public health efforts, while training rural healthcare workers to recognise and manage both mental and physical health conditions will improve access to comprehensive care.

## Additional material


Online Supplementary Document
