# Supplementary material for: The burden of common mental disorders and their association with diabetes in rural Bangladesh: findings from a population-based cross-sectional study
Source: J Glob Health. 2025 Aug 8;15:04220. doi: 10.7189/jogh.15.04220 (PMC12333571; doi:10.7189/jogh.15.04220)
Supplement: Online Supplementary Document [file jogh-15-04220-s001.pdf]

Supplement to: Pires M, King C, Shaha S, Kuddus A, Ahmed N, Abdul Kuddus, Morrison J, Copas A, Ahmed SAU, Nahar T, Haghparast-Bidgoli H, Azad K, Fottrell E. The burden of common mental disorders and their association with diabetes in rural Bangladesh: findings from a population-based cross-sectional study. J Glob Health. 2025;15:04220.

Table S1: ***Defining Sociodemographic Variables***

**Sex**	Male	*Binary variable*
	Female	*Binary variable*
**Age**	>=30	*Continuous variable*
**Marital Status**	Married	Currently Married -*categorical variable*
	Not Married	Divorced, never married, widowed - *categorical variable*
**Income Tertile**	Poorest	Derived from principal component analysis of household assets and household construction - *categorical variable*
	Poor	
	Least Poor	
**Occupation**	Has occupation	Day labourer, rickshaw or van puller, paid domestic worker, fisherman, industrial worker, carpenter, tailor, driver, farmer, service, teacher, banking finance, home-based work, village doctor, priest- *binary variable*
	Does not have formal occupation	Housewife or not working - *binary variable*
**Education**	Education	Attended but did not complete primary school, completed primary school, secondary school, higher education - *binary variable*
	No Education	No formal education or incomplete primary education - *binary variable*

Table S2. ***Weighted crude and adjusted odds ratios for association of T2DM and sociodemographic variables with anxiety***

Variable	Total n	n (%)	Crude OR	p-value	Adjusted OR	p-value
**Diabetic Status**						
No Diabetes	1137	48 (3.8%)	Ref		Ref	
Diabetes	242	12 (5.1%)	1.39 [0.82–2.34]	0.194	1.38 [0.87–2.19]	0.156
**Sex**						
Male	516	13 (2.5%)	Ref		Ref	
Female	876	47 (4.9%)	2.00 [1.10–3.65]	0.028	3.34 [1.30–8.61]	0.017
**Age (continuous)**	1392	—	1.03 [1.01–1.06]	0.017	1.04 [1.02–1.07]	0.006
**Marital Status**						
Married	1182	43 (3.5%)	Ref		Ref	
Not Married	210	17 (7.2%)	2.15 [0.80–5.76]	0.115	1.00 [0.37–2.65]	0.991
**Income Tertile**						
Poorest	465	19 (3.5%)	Ref		Ref	
Poor	463	23 (4.9%)	1.41 [0.86–2.32]	0.157	1.41 [0.86–2.30]	0.156
Least Poor	464	18 (3.4%)	0.97 [0.38–2.50]	0.946	0.87 [0.26–2.85]	0.794
**Occupation**						
Has Occupation	467	12 (2.6%)	Ref		Ref	
No Occupation	925	48 (4.6%)	1.80 [0.79–4.09]	0.146	0.67 [0.24–1.91]	0.418
**Education**						
Education	568	20 (3.5%)	Ref		Ref	
No Education	824	40 (4.3%)	1.22 [0.60–2.47]	0.555	0.68 [0.26–1.82]	0.413

Adjusted for the other factors in the table. Total n observations =1392. #13 missing.

Table S3: ***Association between T2DM and Anxiety when Stratified by Sex***

Non-stratified			
**n=1379**	**Anxiety**	**No Anxiety**	**aOR [95%CI] p-value**
**Diabetes**	n=12 (5.1%)	n=230 (94.9%)	1.38 [0.87, 2.19] p=0.156
**No Diabetes**	n=48 (3.8%)	n=1089 (96.2%)	
**Male**			
**n=514**	**Anxiety**	**No Anxiety**	**aOR [95%CI] p-value**
**Diabetes**	n=3 (4.0%)	n=81 (96.0%)	1.25 [0.426, 3.67] p=0.656
**No Diabetes**	n=10 (2.2%)	n=420 (97.8%)	
**Female**			
**n=865**	**Anxiety**	**No Anxiety**	**aOR [95%CI] p-value**
**Diabetes**	n=9 (5.8%)	n=149 (94.2%)	1.19 [0.85,1.67] p=0.275
**No Diabetes**	n= 38 (4.8%)	n=669 (95.2%)	

Adjustments were maintained for age, income, education, occupation, and marital status.

Did not show evidence of interaction.

Table S4: ***Weighted crude and adjusted odds ratios for association of T2DM and sociodemographic variables with comorbid depression & anxiety***

Variable	Total n	n (%)	Crude OR	p-value	Adjusted OR	p-value
**Diabetic Status**						
No Diabetes	1137	24 (2.0%)	Ref		Ref	
Diabetes	242	9 (3.6%)	1.86 [1.06–3.26]	0.033	1.99 [1.13–3.50]	0.022
**Sex**						
Male	516	6 (1.2%)	Ref		Ref	
Female	876	27 (2.9%)	2.38 [1.47–3.86]	0.002	2.90 [0.62–13.58]	0.156
**Age (continuous)**	1392	—	1.05 [1.01–1.08]	0.009	1.05 [1.01–1.10]	0.015
**Marital Status**						
Married	1182	20 (1.7%)	Ref		Ref	
Not Married	210	13 (5.8%)	3.65 [1.18–11.36]	0.029	1.36 [0.38–4.80]	0.603
**Income Tertile**						
Poorest	465	10 (1.8%)	Ref		Ref	
Poor	463	13 (2.8%)	1.64 [0.65–4.13]	0.266	1.74 [0.62–4.87]	0.261
Least Poor	464	10 (2.1%)	1.19 [0.18–7.69]	0.840	1.08 [0.11–10.88]	0.943
**Occupation**						
Has Occupation	467	4 (1.0%)	Ref		Ref	
No Occupation	925	29 (2.8%)	2.87 [0.87–9.47]	0.078	1.04 [0.16–6.68]	0.961
**Education**						
Education	568	9 (1.6%)	Ref		Ref	
No Education	824	24 (2.6%)	1.60 [0.69–3.72]	0.244	0.77 [0.18–3.27]	0.700

Adjusted for the other factors in the table. Total n observations =1392. #13 missing

Table S5: Association between Comorbid Depression and Anxiety and Diabetes when Stratified by Sex

Non-stratified			
**n=1379**	**Depression & Anxiety**	**No Depression & Anxiety**	**aOR [95%CI] p-value**
**Diabetes**	n= 9 (3.6%)	n=233 (96.4%)	1.99 [1.13, 3.50] p=0.022
**No Diabetes**	n=24 (2.0%)	n= 1113 (98.0%)	
**Male**			
**n=514**	**Depression & Anxiety**	**No Depression & Anxiety**	**aOR [95%CI] p-value**
**Diabetes**	n=2 (0.26%)	n=82 (97.4%)	1.59 [0.25-10.13] p=0.592
**No Diabetes**	n=4 (1.0%)	n=426 (99.1%)	
**Female**			
**n=865**	**Depression & Anxiety**	**No Depression & Anxiety**	**aOR [95%CI] p-value**
**Diabetes**	n=7 (4.2%)	n=151 (96.8%)	1.78 [0.99-3.19] p=0.054
**No Diabetes**	n=20 (2.6%)	n=687 (97.4%)	

Adjustments were maintained for age, income, education, occupation, and marital status.

Did not show evidence of interaction.
